# Large-Format Histology in Diagnosing Breast Carcinoma

**DOI:** 10.1155/2012/618796

**Published:** 2012-12-27

**Authors:** Tibor Tot, Vincenzo Eusebi, Julio A. Ibarra

**Affiliations:** ^1^Department of Pathology and Clinical Cytology, Central Hospital, Uppsala University, 791 82 Falun, Sweden; ^2^Sezione Anatomia, Istologia e Citologia Patologica “Marcello Malpighi”, Università di Bologna, Ospedale Bellaria, 40139 Bologna, Italy; ^3^Department of Pathology, Orange Coast Memorial Medical Center and University of California at Irvine, Irvine, CA 92617, USA

This special issue represents a unique effort to review the experience with this beneficial technique in different pathology laboratories from the United States, Canada, and Europe. Although large-format histology was the topic of a few monographs and has also been discussed in individual reports in a number of journal articles, this special issue is the first to collect knowledge and experience with this technique as used in routine diagnostic histopathology. 

The authors of the first paper by L. Tabár et al. underline the necessity of detailed and systematic correlation of the radiological and pathological findings in diagnosing breast carcinoma and also the need for adequate histology techniques for achieving this correlation. They illustrate how thin and thick large-format histology technique can contribute to achieving such correlation. M. P. Foschini et al. widen the indication for using large-format slides from breast pathology to the pathology of other organs in their beautifully illustrated paper. Tot presents a unique series of 1000 consecutive breast cancer cases worked up with detailed and systematic radiological—pathological correlation and documented in large-format histology slides. The study illustrated the complex morphology of breast carcinoma, where the majority of the cases have multifocal or diffuse in situ or invasive components. M. R. Foster et al. calls attention to the unexpected findings of potential clinical significance which may remain occult if conventional histology technique is used instead of large-format histology. They have evidenced such findings in 26% of their case series. J. A. Ibarra reports on his unique experience with combining standard sections and large-format sections to assess surgical specimens removed following neoadjuvant chemotherapy in a community hospital in the USA, which is an increasingly important issue in every pathology laboratory diagnosing breast carcinomas. In a comprehensive contribution to this special issue, F. L. Tucker summarizes his experience with introducing imaging-assisted large-format histopathology in a pathology department. He demonstrates the detailed program of this process, the rationale and the development in a non-profit health system in the United States. He also reports on clear advantages of the large-format technique used in context with magnetic resonance imaging, compared to conventional histology without such guidance. S. Hofmeyer et al., a research team from a laboratory with 30 year experience in using large-format histology in Falun, Sweden, presents interesting results of a scientific study comparing the subgross morphology of invasive ductal and invasive lobular carcinomas of the breast. One of the highlights of the special issue is the paper demonstrating 3D pathology volumetric technique for calculating breast tumor volume from digitalized whole-mount serial large-format sections by G. M. Clarke et al., a research team from Toronto. As similarities between breast cancer and prostate cancer in both morphology and technical requirements for adequate histological analysis are obvious, the special issue also includes the paper of R. Montironi et al. on total embedding of radical prostatectomy specimens and using large-format histology.

Despite the distance between the laboratories and the differences in working conditions, the experience and expert opinions reported in the papers of this special issue is consistent in evidencing advantages of the large-format histopathology method over the traditional small-block sampling method both as a substitute for standard histopathology but also as an adjunctive tool. The unconcealed ambition of the authors, the publisher, and the editors is to, by providing this evidence, influence the members of the pathology community to accept this method and introduce it in their own everyday practice.

*Tibor Tot* *Tibor Tot* *Vincenzo Eusebi* *Vincenzo Eusebi* *Julio A. Ibarra* *Julio A. Ibarra*

